# Clinical Value of Virtual Reality versus 3D Printing in Congenital Heart Disease

**DOI:** 10.3390/biom11060884

**Published:** 2021-06-14

**Authors:** Ivan Lau, Ashu Gupta, Zhonghua Sun

**Affiliations:** 1 Discipline of Medical Radiation Science, Curtin Medical School, Curtin University, Perth, WA 6102, Australia; i.lau3@postgrad.curtin.edu.au; 2 Department of Medical Imaging, Fiona Stanley Hospital, Perth, WA 6150, Australia; Ashu.Gupta@health.wa.gov.au; 3 Curtin Health Innovation Research Institute (CHIRI), Faculty of Health Sciences, Curtin University, Perth, WA 6102, Australia

**Keywords:** 3D printing, virtual reality, congenital heart disease, diagnosis, visualization

## Abstract

Both three-dimensional (3D) printing and virtual reality (VR) are reported as being superior to the current visualization techniques in conveying more comprehensive visualization of congenital heart disease (CHD). However, little is known in terms of their clinical value in diagnostic assessment, medical education, and preoperative planning of CHD. This cross-sectional study aims to address these by involving 35 medical practitioners to subjectively evaluate VR visualization of four selected CHD cases in comparison with the corresponding 3D printed heart models (3DPHM). Six questionnaires were excluded due to incomplete sections, hence a total of 29 records were included for the analysis. The results showed both VR and 3D printed heart models were comparable in terms of the degree of realism. VR was perceived as more useful in medical education and preoperative planning compared to 3D printed heart models, although there was no significant difference in the ratings (*p* = 0.54 and 0.35, respectively). Twenty-one participants (72%) indicated both the VR and 3DPHM provided additional benefits compared to the conventional medical imaging visualizations. This study concludes the similar clinical value of both VR and 3DPHM in CHD, although further research is needed to involve more cardiac specialists for their views on the usefulness of these tools.

## 1. Introduction

Due to the complex cardiac anatomy and the spectrum of pathologies associated with different types of congenital heart disease (CHD), a complete and scrupulous understanding of the morphology of CHD and the patient’s management is often deemed challenging [1,2,3,4]. Although two-dimensional (2D) and three-dimensional (3D) rendering of the medical imaging datasets allow presentation of extra- and intracardiac structures, they are still limited to be viewed on 2D screens which do not depict the depth of the objects, and hence do not realistically convey the 3D views of anomalies. The interpretation of the heart patho-morphology still relies on operators’ imagination to some extent [2,4,5,6,7,8,9,10,11,12,13].

The limitations of current visualization techniques have driven clinicians and researchers to continually search for solutions to enhance the visualization of complex CHD morphologies. Virtual reality (VR) provides simulation of the real world and allows users to interact directly with the virtual space [14]. The ability of VR to provide the end users free-form 3D visualization in a fully immersive environment has earned it an increasing role in facilitating group diagnostic discussions, complementing conventional surgical planning methods for cardiac surgeries, as well as improving the learning experience among clinicians and trainees [12,13,14]. A study by Ong et al. has demonstrated the value of VR in facilitating group based collaborative discussion, aiding the preoperative planning process for CHD surgeries [14]. Kim et al. further validated this by evaluating the usefulness of full-immersive VR in facilitating group diagnostic discussions compared to that of nonimmersive VR and a conventional 2D display. The result suggests that full-immersive VR is not only the most preferred display system among the participants, it also significantly improves the diagnostic accuracy of group discussions [12].

3D printing technology is another option that has attracted increasing interest in cardiovascular medicine by providing more comprehensive visualization of the anomalous heart [1,2,3,4,5,6,7,8,9,10,15,16]. The main reason for using 3D printed models is to overcome the limitations of 2D medical images which fail to fully demonstrate the spatial relationships between intracardiac structures as well as the geometric relationship between the great vessels and surrounding anatomies [17,18,19]. With the tangible 3D printed model in hand, physicians can manipulate and assess the diseased heart to their liking. A multicenter study involving 40 patients with complex CHD reported the usefulness of 3D printed heart models (3DPHM) in the preoperative planning of their surgeries. In 19 of the 40 cases, the 3DPHM helped the surgeons to redefine a more suitable surgical approach through improving their perception of the size and spatial relationship of cardiac structures [15].

However, both the use of VR in medicine and application of 3DPHM in CHD surgeries are still in their infancy. To the best of our knowledge, there is no published article to compare the value of VR against 3DPHM in providing a more comprehensive visualization for educating young physicians or medical practitioners about CHD and even aiding cardiac specialists in preoperative planning of CHD. Thus, the aim of this study was to compare the clinical value of both VR and 3D printing in diagnostic assessment, medical education, and preoperative planning of CHD through subjective evaluations from medical practitioners.

## 2. Materials and Methods

### 2.1. Study Design

**Figure 1:**
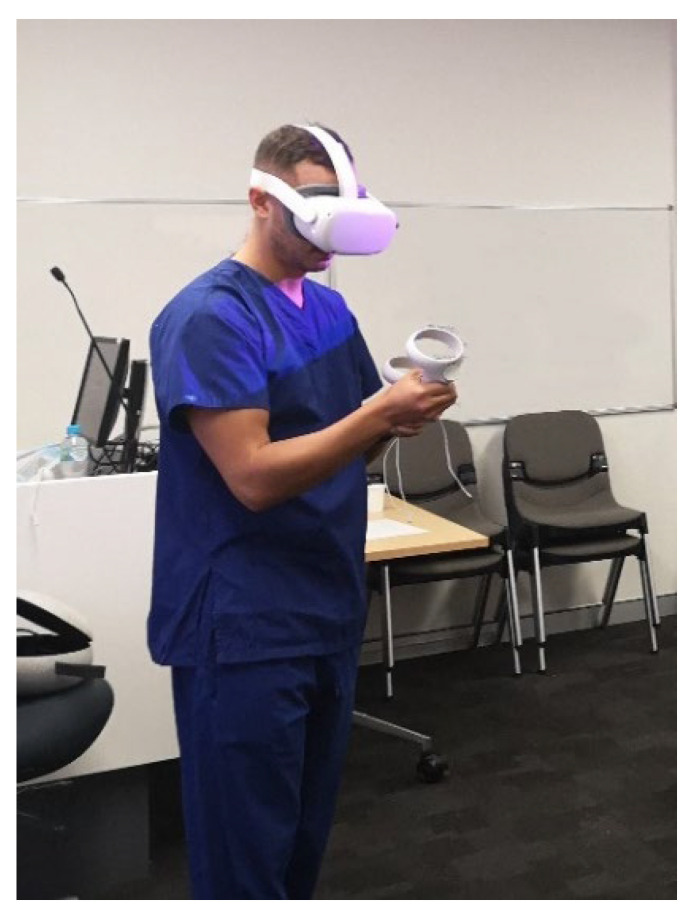
One of the participants assessed the VR heart models using Oculus Quest 2 (Facebook Technologies, LLC, Irvine, California, United States). He was able to interact with the VR heart models using hand-held controllers.

A cross-sectional study was conducted to compare the clinical value of both VR and 3DPHM. The study was approved by the Curtin University Human Research Ethics Committee. To recruit the study participants, the study was advertised in the radiology department of a major public hospital in Perth, Western Australia. Thirty-five participants comprising radiologists, sonographers, and radiographers voluntarily participated in the study. Due to the limited number of VR headsets, the participants were divided into groups of 3 or 4 to ensure each of them had sufficient time for VR and 3DPHM assessment. Each group attended a 15-min VR and 3DPHM demonstration session. At the start of the session, they were briefed with the process of conversion from medical imaging datasets to VR and 3DPHM (an average of 3 min). Following that, they were given basic training of how to interact with the VR models using hand-held controllers. For the rest of the session (an average of 10 min), all the participants were encouraged to assess both VR and 3DPHM closely (Figure 1). There was no restriction in terms of the model assessment. The participants were allowed to assess the VR and 3DPHM in the order that they preferred, and were allowed discussion. At the end of the demonstration, they were asked to fill out a questionnaire, which was designed primarily to address the following aspects: (i) degree of realism of the heart models for each technology; (ii) the ability of each technology in displaying pathology and anatomy; (iii) the utility of each technology in educating medical students or young physicians about complex CHD; (iv) the usefulness of each technology in preoperative planning for CHD. The questionnaire can be found in Supplementary file S1. All responses were recorded anonymously.

### 2.2. Generation of the Heart Models

The computed tomography angiography (CTA) imaging datasets of four different CHD cases were collected and used as the source data for this project. These four cases featured a range of CHD with different levels of complexity, defined by the Aristotle Basic Complexity Level (ABCL): atrial septal defect (ASD, ABCL = 1), ventricular septal defect (VSD, ABCL = 2), Tetralogy of Fallot (ToF, ABCL = 3), and double outlet right ventricle (DORV, ABCL = 4).

**Figure 2:**
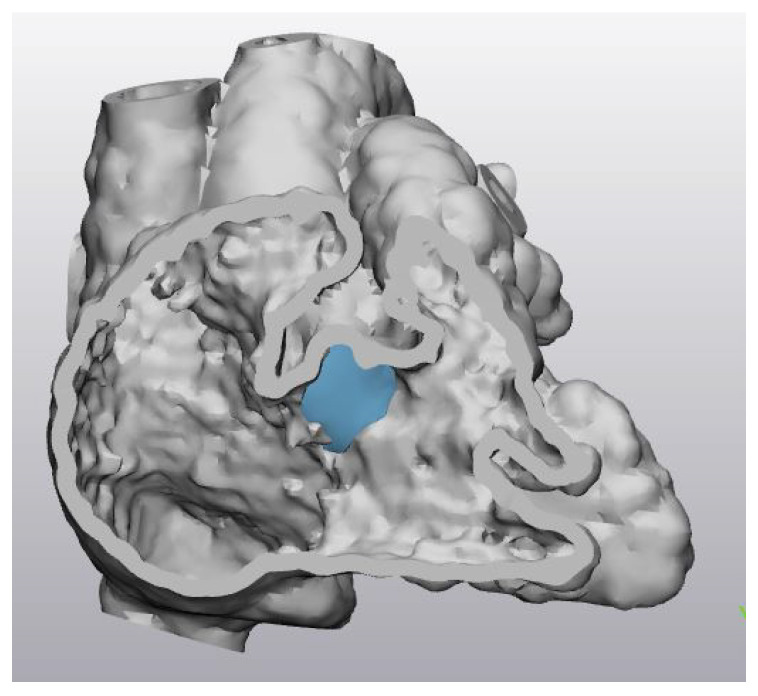
A transected digital heart model demonstrating a ventricular septal defect with a virtual patch (in blue) over the defect.

In order to convert the imaging dataset into printable and VR-viewable files, the heart was segmented to separate it from the surrounding bones, organs, and tissues. This process was performed using Mimics Innovation Suite 23.0 (Materialise HQ, Leuven, Belgium). An arbitrary thickness of a 1mm-thick shell was added onto the digital model before it was hollowed out to prevent the 3D printed model from collapsing during the 3D printing process. Following that, the digital model was smoothed out to remove any tiny spikes on the heart surface using 3-Matic, which is 3D modelling software accompanied by the Mimics Innovation Suite (Materialise HQ, Leuven, Belgium). A cutting plane transecting the right atrium and right ventricle was also created to separate the models in halves in order to demonstrate the critical anatomy (Figure 2). This cutting plane was kept consistent for both VR and 3DPHM.

For each case, a virtual patch was designed using 3-Matic to provide an option for the users to view the heart when the defect was closed (Figure 2).

### 2.3. 3D Printing of the Heart

For 3D printing purposes, the digital heart models were converted into a standard tessellation language (STL) format. The models were printed in polyurethane (TPU80A, Fabbxible Technology Sdn Bhd, Pulau Pinang, Malaysia) and flexible resin (Flexible V4 Resin, Formlabs, Somerville, Massachusetts, United States), both with shore hardness of 80A.

### 2.4. Creation of the VR Project

Unity 3D (Unity Technologies, San Francisco, California, USA) was used with C# coding to build a VR project to allow users to grab the heart models, rotate, and to view them up close within an immersive, virtual environment. The digital heart models were loaded into Unity 3D in object file (OBJ) format. Four tables were designed and placed in the virtual scene in Unity 3D, with the heart models of four different cases placed on the table respectively. Heart models with the virtual patch were also loaded into the virtual scene so that the users could visualize how the heart appeared when the defect was closed. The VR build was then exported in an android (APK) file format and loaded into Oculus Quest 2 (Facebook Technologies, LLC, Irvine, California, United States). Within the VR project, the users could turn around and use the hand-held controllers to grab different heart models, rotate them “in hand”, and view them up close (Figure 1).

**Figure 3:**
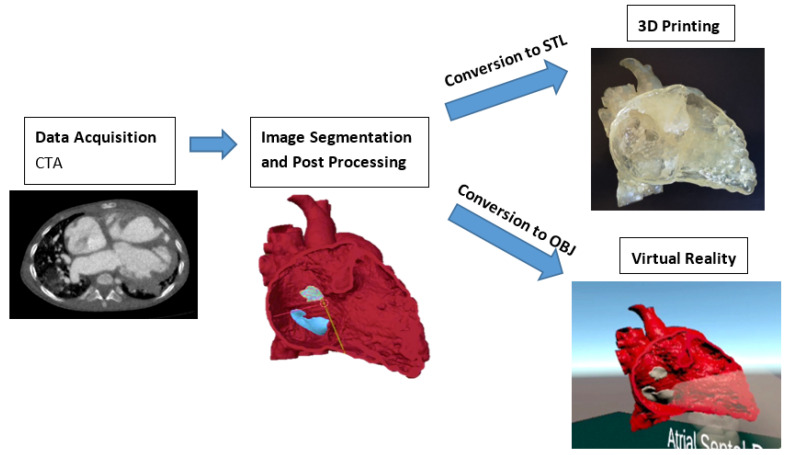
Steps involved in the creation of 3DPHM and the VR project. 3D, three-dimensional; CTA, computed tomography angiography; OBJ, object files; STL, standard tessellation language.

Figure 3 illustrates the steps involved in creating the 3DPHM and the VR project. The video and images of VR and 3DPHM can be found in Supplementary files S2 and S3.

### 2.5. Statistical Analysis

The data from the questionnaire were analyzed using SPSS 26.0 (IBM SPSS statistics). Quantitative data and categorical variables were analyzed using descriptive statistics (frequencies and median). Kendall’s W (coefficient of concordance) test was used to assess the agreement among participants with regard to the ability of VR and 3DPHM in demonstrating anatomy and pathology. The Mann-Whitney U test was used to compare the ratings given for both VR and 3DPHM, and also for subgroup (doctors and non-doctors) analysis. A *p*-value below 0.05 was considered statistically significant. Free-text in open-ended questions was analyzed using thematic analysis.

## 3. Results

Out of the 35 participants, 6 participants did not fully complete the questionnaire, therefore their responses were excluded from the data analysis. As a result, a total of 29 responses were included in the data analysis (1 cardiac radiologist, 1 interventional radiologist, 3 general radiologists, 4 radiology registrars, 3 sonographers, 16 radiographers, and 1 student radiographer).

Table 1, Table 2, Table 3 and Table 4 are the frequency tables of the individual questions on the questionnaire with regard to the four aspects. Generally, both VR and 3DPHM were comparable with each other in terms of the degree of realism (Table 1); the 3DPHM was better in displaying the CHD pathology and anatomy (Table 2); VR was perceived as more useful for educating medical students and young physicians about CHD (Table 3), as well as preoperative planning (Table 4). The Kendall’s W test shows no significant difference in the frequencies between VR and 3DPHM according to the results shown in Table 2 (*p* = 0.32), but there was a significant difference between other comparisons (VR vs. Both and Unsure, 3DPHM vs. Both and Unsure, *p* = 0.04).

The Mann–Whitney U test shows no significant difference between VR and 3DPHM in terms of the ratings for their usefulness in medical education and preoperative planning, with the mean rank of 3DPHM slightly higher than the VR models in both aspects (*p* = 0.35 and *p* = 0.54, respectively) (Table 5). Subgroup analysis was also performed to determine if there was any difference in the responses depending on the participants’ clinical background. The clinicians were grouped into doctors’ and non-doctors’ groups for the subgroup analysis. The non-doctors’ group consisted of sonographers, radiographers, and a student radiographer (*n* = 20), while the others were grouped as the doctors’ group (*n* = 9). The Mann–Whitney U test demonstrates no significant difference in responses between the doctors’ and non-doctors’ groups (Table 6).

**Figure 4:**
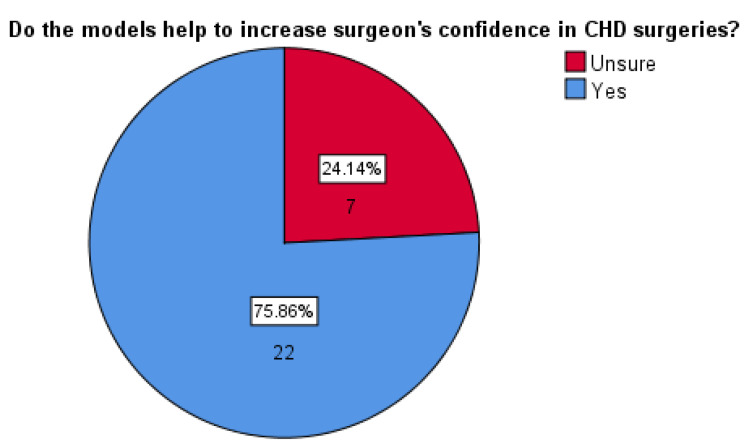
Responses from the participants with regard to their opinion on whether VR and 3DPHM help to increase surgeons’ confidence in CHD surgeries.

Out of the 29 participants, 22 (76%) indicated both the VR and 3DPHM were helpful to increase surgeons’ confidence for CHD surgeries (Figure 4). No one indicated ‘No’ for this question, suggesting the participants’ positivity towards this aspect.

Twenty-one participants (72%) indicated both the VR and 3DPHM provided additional benefits compared to the conventional medical imaging visualizations. Eight of them commented on the 3D visualization helping them to better appreciate the spatial information; six of them indicated the tactile models (3DPHM) as being beneficial for the learning of CHD; three of them pointed out both of the technologies as being helpful in visualizing and conceptualizing the cardiac structures; and four of them mentioned the added benefits of the models to convey anatomy or pathology to patients.

## 4. Discussion

This study presents a side-by-side comparison of both 3D printing and VR technologies from subjective evaluations of a group of medical practitioners. The results show both of them being on a similar level with each other in providing a better visualization experience compared to the conventional visualization technique. In fact, the advent of 3D printing and VR are currently transforming and improving visualization techniques for medical imaging [12,20,21]. The benefits of 3D printing in preoperative planning for CHD can be seen from the increasing reports over the years [1,2,3,4,5,6,7,8,9,10,15,16,17,18,19,22]. Yoo et al. reported the use of 3DPHM in planning and simulation of an extremely complex heart surgery for dextrocardia which involved heart transplantation [22]. In another cross-sectional study which involved 71 pediatric cardiologists from different countries, it was found that 85% of the participants agreed or strongly agreed, that 3DPHM are beneficial in the treatment of CHD, with surgical planning as the primary utility of 3DPHM [23]. This echoes the finding from a recent meta-analysis, which found preoperative planning being the most relevant application of 3DPHM [24]. VR has also been reported in the current literature for its ability to provide an immersive, interactive, and free-form visualization experience, despite it not being tactile like 3DPHM [12,14,25,26,27]. Unlike 3DPHM, being static and unable to show cardiac functional information, the VR project can be “programmed” to show dynamic cardiac models [28,29], to allow users to scale, rotate, crop the cardiac models, and change the viewing planes according to their needs [12].

However, both of these technologies do not come without limitations. The main barrier that impedes the wide application of 3D printing in CHD is the associated start-up cost. This includes the costs of 3D printers, their operation and maintenance, 3D printing materials, segmentation software, and the hiring of expertise [23,30]. With the advancement of this technology, this cost is expected to be reduced [20]. A recent study reported that the low-cost 3DPHM is just as accurate as the high-cost 3DPHM in delineating cardiac anatomy [31]. Therefore, depending on the medical application, a low-cost 3DPHM can be used to reduce the cost of 3D printing. Secondly, the segmentation process is laborious and time-consuming. It requires skilled personnel or most often, a multi-disciplinary team to have the knowledge of anatomy, imaging physics, and engineering knowledge pertaining to 3D printing [23,30]. As for VR, the main limitation is the need to wear bulky headsets [32]. Additionally, the users may also have motion sickness, which could pose risks to patients’ safety during procedures [21,32].

In the present study, the results demonstrated that 3DPHM are perceived to be better in displaying CHD pathology and anatomy. This contradicts the finding of another study. Raimondi et al. compared 3DPHM and VR models of three cases of complex CHD, and their result suggested that the VR models were superior to the 3DPHM in conveying ventricular-arterial connections and the aortic arch [25]. This could be related to the difference in the development of VR models. Raimondi et al. used DIVA software to convert the DICOM dataset into VR models in a matter of minutes, skipping the image segmentation step completely. This software exploits the potential of volumetric rendering, which is able to generate 3D models without the need of image segmentation [33]. The software also allowed the end users to change the viewing plane and navigate within the heart [25]. This is different to the VR project that is presented in our study. Our VR project is relatively simple to create and easy to use, which allows users to get the hang of it really quickly (Supplementary file S2). The users were allowed to grab, rotate and view the VR models up close by simply bringing the hand controller closer to them. It also supports multiple cardiac models to be loaded within the VR project. However, the users were not able to change the viewing planes or crop the cardiac models, which is one of the limitations of the presented VR project. The simplicity of this VR project makes it quite different from other studies in which the users are able to change the clipping plane [12,14,25,26], or view the models intraluminally [27]. In fact, there is no standardized way for creating a VR project [25]. The users can design it according to their needs. Even though this VR project does not feature as many interactive elements as other studies reported, its ability to provide a fully immersive and enhanced viewing experience should not be overlooked. During the evaluation, one of the participants dropped the hand controller as she was putting the cardiac model back onto the virtual table, thinking that there was a ‘real’ table in front of her. This implicitly highlights the immersiveness of the VR tool.

There is one contradictory finding from the results, which could be related to the insufficiency of the design of the questionnaire. While the majority of the participants indicated VR is more useful in medical education and preoperative planning, 3DPHM has a higher mean rank compared to VR according to the ratings given by the participants, although the difference in mean ranks does not reach statistical significance. This can be explained by the limitation of the use of a 5-point rating scale in this instance, and it might be inadequate to measure the difference in responses. Many of the participants have given both VR and 3DPHM the same ratings even though they indicated otherwise for questions in Table 3 and Table 4.

There are a few limitations in this study which need to be acknowledged. Firstly, this study only involves radiologists and radiographers; therefore, the application of 3D printing and VR in CHD may not be as relevant for them as opposed to cardiac specialists. Secondly, there were only 15 min for the evaluations of both VR and 3DPHM, which may not be sufficient for some participants should they need a longer time to get a better understanding of these two new visualization tools. Thirdly, as the participants were allowed to assess the models freely in any order with group discussions permitted, their responses could have been affected by the order effect and conformity bias. Lastly, we only included four categories of CHD in this study, which limits it to CHD cases only. Further studies should look at the application of VR and 3DPHM in other cardiovascular disease, such as complex aortic aneurysm or aortic dissection.

## 5. Conclusions

This study compared the clinical value of both VR and 3D printing in diagnostic assessment, medical education, and preoperative planning of CHD through subjective evaluations from medical practitioners. The results show no significant difference between both technologies in the aforementioned areas. However, we should not overlook the benefits of both technologies in providing advanced visualization in medicine. Future studies should involve cardiac specialists to provide more pertinent opinions and evaluations of these two visualization tools in CHD and other cardiovascular diseases. Additionally, it could be useful for future studies to investigate the difference in the level of clinical benefits gained from VR and 3DPHM for CHD of different complexities.

## Figures and Tables

**Table 1:** Participants’ responses on the degree of realism of the VR and 3DPHM.

	Option	VR Models		3DPHM		Both are the Same		Unsure	
Question		Freq.	%	Freq.	%	Freq.	%	Freq.	%
Better depth perception?		8	27.6	16	55.2	5	17.2	0	0
Better and more comprehensive viewing experience?		19	65.5	8	27.6	1	3.4	1	3.4
More realistic visualization?		9	31.0	12	41.4	5	17.2	3	10.3
Total		36	41.38	36	41.38	11	12.64	4	4.60

3DPHM, 3D printed heart models; Freq., frequency; VR, virtual reality.

**Table 2:** Participants’ responses on the ability of VR and 3DPHM in displaying pathology and anatomy.

	Option	VR Models		3DPHM		Both are the Same		Unsure	
Question		Freq.	%	Freq.	%	Freq.	%	Freq.	%
Better appreciation of heart defects?		14	48.3	11	37.9	3	10.3	1	3.4
Better understanding of the spatial relationship between cardiac structures?		7	24.1	17	58.6	5	17.2	0	0
Better visualization of external cardiac structures?		5	17.2	19	65.5	4	13.8	1	3.4
Better visualization of internal cardiac structures?		15	51.7	13	44.8	1	3.4	0	0
Total		41	35.35	60	51.72	13	11.21	2	1.72

3DPHM, 3D printed heart models; Freq., frequency; VR, virtual reality.

**Table 3:** Participants’ responses on the utility of VR and 3DPHM in educating medical students or young physicians about complex CHD.

	Option	VR Models		3DPHM		Both are the Same		Unsure	
Question		Freq.	%	Freq.	%	Freq.	%	Freq.	%
More useful in educating medical students or young physicians about CHD?		12	41.4	4	13.8	12	41.4	1	3.4

3DPHM, 3D printed heart models; Freq., frequency; VR, virtual reality.

**Table 4:** Participants’ responses on the usefulness of VR and 3DPHM in preoperative planning for CHD.

	Option	VR Models		3DPHM		Both are the Same		Unsure	
Question		Freq.	%	Freq.	%	Freq.	%	Freq.	%
More useful in preoperative planning for CHD?		10	34.5	9	31.0	4	13.8	6	20.7

3DPHM, 3D printed heart models; Freq., frequency; VR, virtual reality.

**Table 5:** Participants’ responses on the ratings for VR and 3DPHM ^a^.

	Option	VR Models, *n* = 29 Clinicians	3DPHM, *n* = 29 Clinicians	Mann-Whitney U-Value	*p*-Value
Question					
Rate the usefulness of the models in medical education		4 (27.60)	4 (31.40)	365.50	0.35
Rate the usefulness of the models in pre-operative planning		4 (28.26)	4 (30.74)	384.50	0.54

3DPHM, 3D printed heart models; VR, virtual reality. ^a^ Data are median score (mean rank).

**Table 6:** Subgroup analysis for participants’ responses on the ratings for VR and 3DPHM ^a^.

	Option	Doctors’ Group, *n* = 9	Non-doctors’ Group, *n* = 20	Mann-Whitney U-Value	*p*-Value
Question					
Rate the usefulness of VR models in medical education		4 (11.22)	4 (16.70)	56.00	0.07
Rate the usefulness of 3DPHM in medical education		4 (11.11)	5 (16.75)	55.00	0.07
Rate the usefulness of VR models in pre-operative planning		4 (11.06)	4 (16.77)	54.50	0.07
Rate the usefulness of 3DPHM in pre-operative planning		4 (12.39)	4.5 (16.18)	66.50	0.23

3DPHM, 3D printed heart models; VR, virtual reality. ^a^ Data are median score (mean rank).

## Data Availability

The datasets used during the current study are not publicly available due to strict requirements set out by the Human Ethics Research Committee regarding the storage and use of the data by authorised investigators.

## Supplementary Materials

The following are available online at https://www.mdpi.com/article/10.3390/biom11060884/s1, Supplementary file S1: Questionnaire for medical practitioners, Supplementary file S2: Video of VR project, Supplementary file S3: Images of 3D Printed Heart Models.
