# Supplementary material for: Clinical Value of Virtual Reality versus 3D Printing in Congenital Heart Disease
Source: Biomolecules. 2021 Jun 14;11(6):884. doi: 10.3390/biom11060884 (PMC8232263; doi:10.3390/biom11060884)
Supplement: Supplementary file 1 [file biomolecules-11-00884-s001.zip › biomolecules-1218830-Supplementary files/Supplementary File 3.pdf]

Supplementary File 3: Images of 3D Printed Heart Models

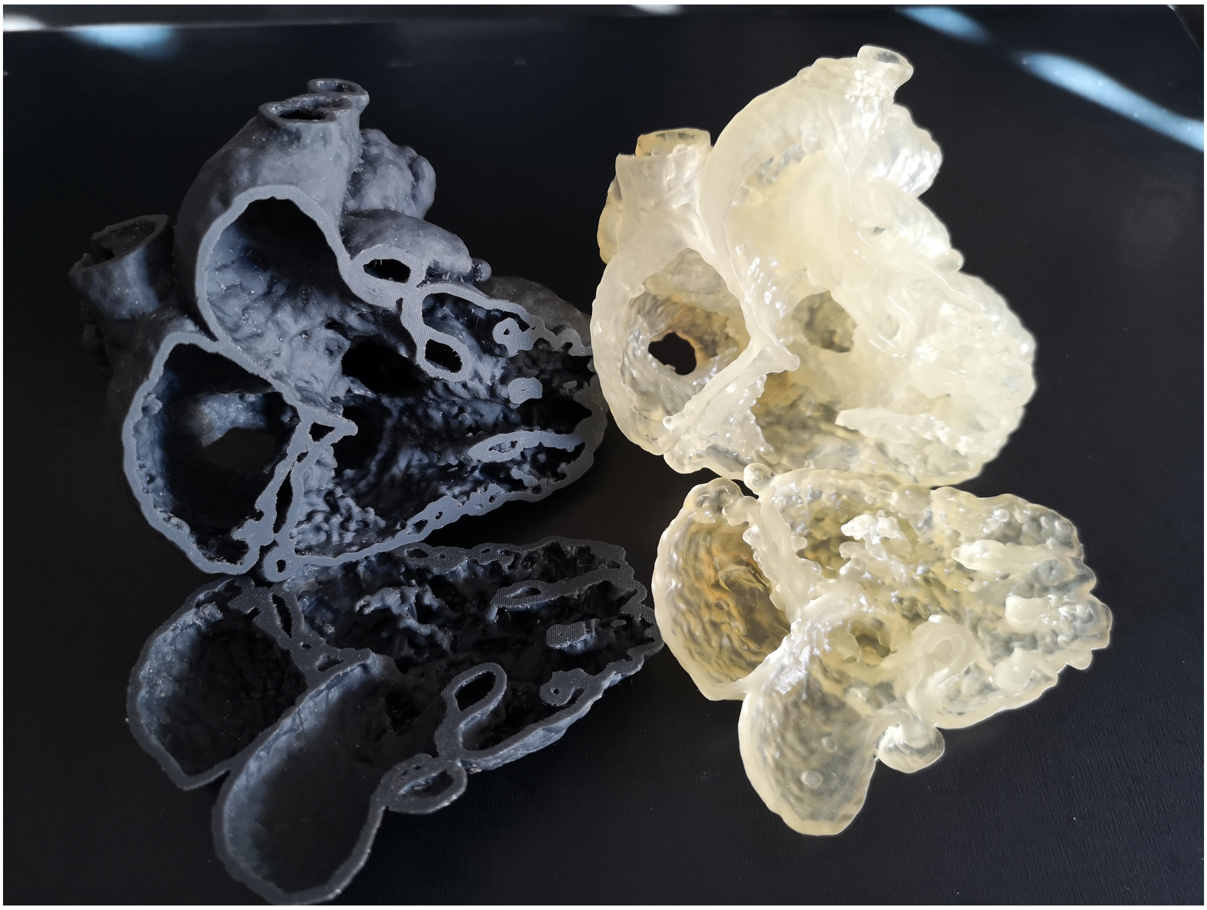

Figure 1: 3D printed heart models in two different materials, Flexible V4 Resin (gray) and TPU 80A (translucent).

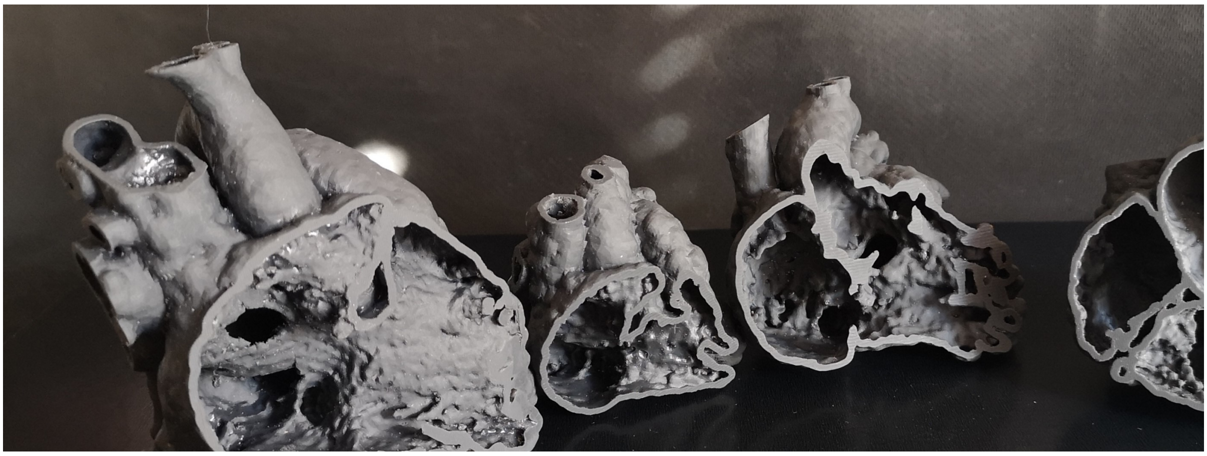

Figure 2: 3D printed heart models of 4 different types of congenital heart disease. Starting from left: atrial septal defect, ventricular septal defect, Tetralogy of Fallot, double outlet right ventricle.
