# Supplementary material for: Clinical Value of Virtual Reality versus 3D Printing in Congenital Heart Disease
Source: Biomolecules. 2021 Jun 14;11(6):884. doi: 10.3390/biom11060884 (PMC8232263; doi:10.3390/biom11060884)
Supplement: Supplementary file 1 [file biomolecules-11-00884-s001.zip › biomolecules-1218830-Supplementary files/VR vs 3D questionnaire R1.docx]

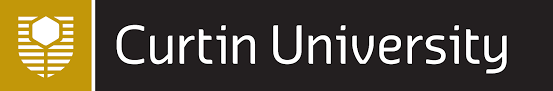
**General information**

**Are you a…**

☐ Cardiologist ☐ Interventional Cardiologist ☐ Cardiac Radiologist ☐ General Radiologist ☐ Radiology Registrar

☐ Radiographer ☐ Others: ___________

**Please place a tick (✓) in the box of your chosen option.**

| **No** | **Questions** | **VR models** | **3D printed heart models** | **Both are the same** | **Unsure** | **None** |
| --- | --- | --- | --- | --- | --- | --- |
| 1 | Which allows a better depth perception for the heart structures? |  |  |  |  |  |
| 2 | Which allows a better and more comprehensive viewing experience for yourself? |  |  |  |  |  |
| 3 | Which has a more realistic visualization compared to 3D rendered DICOM images*? |  |  |  |  |  |
| 4 | Which helps you to appreciate the heart defects better? |  |  |  |  |  |
| 5 | Which helps you to understand the spatial relationship between the cardiac structures better? |  |  |  |  |  |
| 6 | Which allows you to visualise the external cardiac structures better? |  |  |  |  |  |
| 7 | Which allows you to visualise the internal cardiac structures better? |  |  |  |  |  |
| 8 | In your opinion, which is more useful in the aspect of educating medical students or young physicians about congenital heart disease? |  |  |  |  |  |
| 9 | In your opinion, which is more useful in the aspect of pre-operative planning for congenital heart disease surgeries? |  |  |  |  |  |

1. In your opinion, please rate the usefulness of both the VR and 3D printed heart models in educating medical students or young physicians about congenital heart disease. (1-5, 1 = not useful, 5 = extremely useful)

VR models 1 2 3 4 5

3D printed heart models 1 2 3 4 5

1. In your opinion, please rate the usefulness of both the VR and 3D printed heart models in preoperative planning.

(1-5, 1 = not useful, 5 = extremely useful)

VR models 1 2 3 4 5

3D printed heart models 1 2 3 4 5

1. Do you think patient-specific 3D models (regardless of VR or 3D printed) can help to increase the surgeon’s confidence in congenital heart disease surgeries?

☐ Yes ☐ No ☐ Unsure

1. Do you think patient-specific 3D models (regardless of VR or 3D printed) have provided additional benefits compared to the conventional medical imaging visualizations (e.g. 3D CT or MRI image visualization)? If so, briefly explain the reasons.
2. Can you identify any other areas that VR or 3D printed heart models could be useful in clinical practice?
